# Cardiac hydatid cyst in an atypical location presenting with frequent premature ventricle contractions

**DOI:** 10.1093/ehjcr/ytae008

**Published:** 2024-01-09

**Authors:** Nikhil Singhania, Chinmay Parale, Avinash Anantharaj

**Affiliations:** Department of Cardiology, Jawaharlal Institute of Postgraduate Medical Education and Research, JIPMER Campus Road, Dhanvantari Nagar, Puducherry 605006, India; Department of Cardiology, Jawaharlal Institute of Postgraduate Medical Education and Research, JIPMER Campus Road, Dhanvantari Nagar, Puducherry 605006, India; Department of Cardiology, Jawaharlal Institute of Postgraduate Medical Education and Research, JIPMER Campus Road, Dhanvantari Nagar, Puducherry 605006, India

**Figure:**
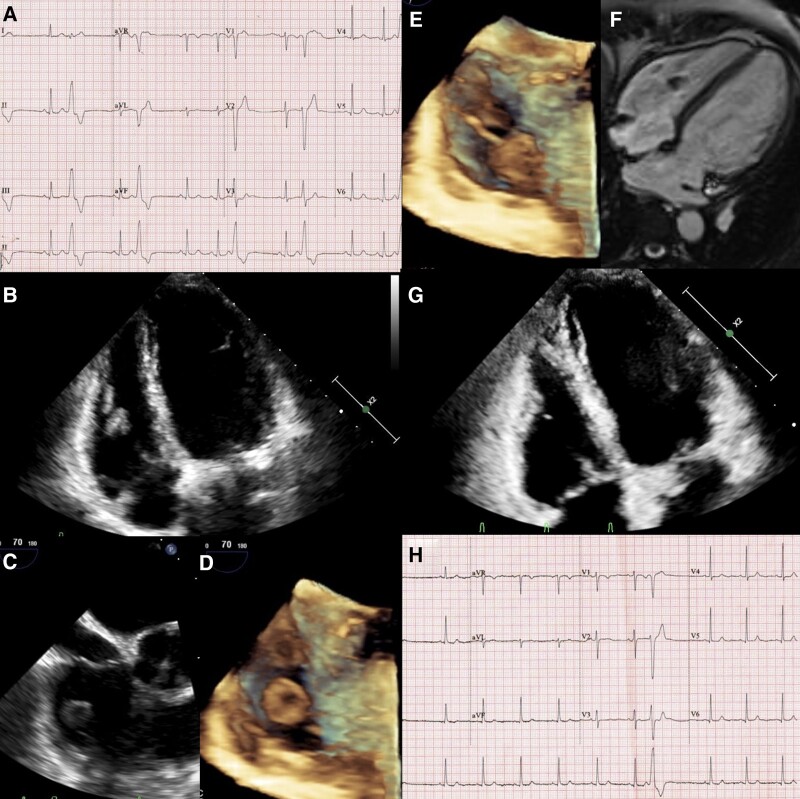


A 44-year-old woman diagnosed with uterine fibroid, admitted for elective vaginal hysterectomy, was referred to us for the evaluation of frequent premature ventricular complexes (PVCs). She had no history of weight loss, diaphoresis, fever, rash, or any cardiac, gastrointestinal, or respiratory symptoms. The PVCs (*Panel A*) were of a left bundle branch abnormality (LBBB) morphology with an inferior QRS axis and a mid-precordial transition—features suggestive of anterior right ventricular outflow tract (RVOT) origin. The 24-h PVC burden was 19%. Transthoracic echocardiography (TTE) showed a mass of 15 × 10 mm, freely oscillating in the right ventricle (*Panel B*). On transoesophageal echocardiography (TEE), the mass was cystic and attached to one of the chordae tendineae with a suspicious stalk (*Panels C–E*; see Supplementary material online, *Videos S1* and *S2*). Biventricular function was normal. On magnetic resonance imaging (MRI), it appeared hypointense, showed no late gadolinium enhancement, and was clearly attached to the papillary muscle via chordae tendineae (*Panel F*; see Supplementary material online, *Video S3*). With a suspicion of hydatid cyst, echinococcal IgG enzyme-linked immunosorbent assay (ELISA) was done and was positive. Contrast-enhanced computed tomography of the thorax and abdomen revealed no additional cysts. Despite the atypical location, the diagnosis of cardiac hydatid cyst was deemed to be highly likely considering the very high specificity of IgG ELISA for the diagnosis of echinococcal infection (98–100%). Considering the patient’s asymptomatic presentation, along with the smaller size and cystic stage of the disease and prior case reports showing successful treatment outcomes using medical therapy alone, the patient was started on extended-release metoprolol succinate (25 mg/day) and albendazole (800 mg/day). After 1 month, her PVC burden had reduced, and TTE showed a significant decrease in the mass size. A second follow-up a month later showed complete resolution of the mass and a decline in PVCs (*Panels G* and *H*) with a 24-h PVC burden of 6%, confirming the diagnosis of cardiac hydatid cyst. The patient completed a total of 12 weeks of therapy to ensure adequate sterilization of the cyst. Follow-up monitoring was continued regularly to detect recurrence.

(*A*) A 12-lead chest electrocardiogram showing frequent right ventricular outflow tract premature ventricular complexes. (*B*) Transthoracic echocardiography apical four-chamber view showing mass in the right ventricle. (*C*) Transoesophageal echocardiography mid-oesophageal view showing cystic nature of mass. (*D*) 3D transoesophageal echocardiography view showing cystic nature of mass. (*E*) 3D transoesophageal echocardiography view showing mass attached to one of the chordae tendineae with a suspicious stalk. (*F*) Cardiac magnetic resonance imaging axial four-chamber view showing hypointense mass attached to the papillary muscle via chordae tendineae. (*G*) Transthoracic echocardiography apical four-chamber view after 4 weeks of treatment showing complete resolution of the mass. (*H*) A 12-lead chest electrocardiogram after 4 weeks of therapy showing decrease in frequency of premature ventricular complexes.

## Supplementary Material

ytae008_Supplementary_Data

## Data Availability

No data were generated or analysed for or in support of this paper.

## Supplementary material

**Consent:** The authors confirm that written consent for submission and publication of this case report, including images and associated text, has been obtained from the patient in line with COPE guidance.

**Funding:** None declared.

